# Cytokinesis in Bloodstream Stage *Trypanosoma brucei* Requires a Family of Katanins and Spastin

**DOI:** 10.1371/journal.pone.0030367

**Published:** 2012-01-18

**Authors:** Corinna Benz, Caroline Clucas, Jeremy C. Mottram, Tansy C. Hammarton

**Affiliations:** Wellcome Trust Centre for Molecular Parasitology, Institute of Infection, Immunity and Inflammation, College of Medical, Veterinary and Life Sciences, University of Glasgow, Glasgow, United Kingdom; Technion-Israel Institute of Technology, Israel

## Abstract

Microtubule severing enzymes regulate microtubule dynamics in a wide range of organisms and are implicated in important cell cycle processes such as mitotic spindle assembly and disassembly, chromosome movement and cytokinesis. Here we explore the function of several microtubule severing enzyme homologues, the katanins (KAT80, KAT60a, KAT60b and KAT60c), spastin (SPA) and fidgetin (FID) in the bloodstream stage of the African trypanosome parasite, *Trypanosoma brucei.* The trypanosome cytoskeleton is microtubule based and remains assembled throughout the cell cycle, necessitating its remodelling during cytokinesis. Using RNA interference to deplete individual proteins, we show that the trypanosome katanin and spastin homologues are non-redundant and essential for bloodstream form proliferation. Further, cell cycle analysis revealed that these proteins play essential but discrete roles in cytokinesis. The KAT60 proteins each appear to be important during the early stages of cytokinesis, while downregulation of KAT80 specifically inhibited furrow ingression and SPA depletion prevented completion of abscission. In contrast, RNA interference of FID did not result in any discernible effects. We propose that the stable microtubule cytoskeleton of *T. brucei* necessitates the coordinated action of a family of katanins and spastin to bring about the cytoskeletal remodelling necessary to complete cell division.

## Introduction

The protozoan parasite, *Trypanosoma brucei*, is the causative agent of the human and animal African trypanosomiases, which are frequently fatal if untreated. It has a digenetic life cycle, replicating in the alimentary canal of its vector, the tsetse fly, and the bloodstream of the mammal. During its life cycle, the trypanosome undergoes a series of differentiation steps yielding forms that differ morphologically and biochemically, as well as with respect to their replicative status [1]. The long slender bloodstream stage and the procyclic and epimastigote forms in the tsetse fly are proliferative; other life cycle stages are either cell cycle arrested (the short stumpy bloodstream stage and the metacyclic form in the tsetse salivary glands), or, in the case of the long proventricular form, undergo a single asymmetric cell division [2], [3]. Considerable differences in cell cycle regulation are known to exist between the long slender bloodstream stage and the procyclic form, and the subcellular organisation of different life cycle stages also varies significantly [3]–[6]. The vermiform morphology of *T. brucei* is conferred by a subpellicular microtubule cytoskeleton [7]. Microtubules within this cytoskeletal corset are arranged in parallel with their plus ends orientated to the posterior of the cell [8]. Microtubule-associated proteins (MAPs) crosslink the microtubules to each other and to the plasma membrane, stabilising the cytoskeleton [7], [9], [10]. During cell division, the cytoskeleton is inherited semi-conservatively; the cytoskeleton as a whole does not break down at any point, and newly forming microtubules interdigitate between existing ones [11]. This therefore imposes a barrier to cell division, since the microtubules and their linking MAPs must be broken and remodelled at the site of cleavage. Cytokinesis in *T. brucei* involves the ingression of a cleavage furrow from the anterior end of the cell to the posterior, following the longitudinal axis of the cell. This is a very divergent mode of division compared to the contractile actomyosin ring used by mammals and yeasts to pinch the cell in two. Indeed, no role for actin or myosin in trypanosome cytokinesis has been uncovered, and instead these proteins are required for endocytosis in the bloodstream stage [12], [13].

Multiple regulators of trypanosome cytokinesis have been identified [14]–[18], but their interplay and targets remain largely unexplored. Moreover, information about the mechanics and effectors of cell division is lacking [6], [14]. Katanin, spastin and fidgetin are members of a family of AAA ATPases (ATPases Associated with various cellular Activities) that influence microtubule dynamics in a variety of organisms [19]–[21]. These enzymes sever microtubules along their length, thus shortening them and increasing the overall number of microtubules, as well as increasing the pool of free tubulin molecules, which can nucleate new microtubules. Katanin is a heterodimer, consisting of a regulatory p80 and a catalytic p60 subunit [22], [23]. The regulatory subunit is important for substrate recognition and targeting of the catalytic subunit, although it is not essential for its activity [24], and p60 katanin can be recruited to mammalian centrosomes by other means [25]. Katanin activity is also regulated by microtubule binding proteins [26]–[28], which can inhibit its activity, and the activities of both katanin and spastin are regulated by post-translational modification of microtubules [27], [29], [30]. Spastin and fidgetin have high homology to katanin p60, but have not been reported to interact with a regulatory subunit. p60 katanin and spastin are known to oligomerise into hexameric rings in the presence of ATP, stimulating their ATPase activity and creating a central pore into which it is thought the C-terminal tail of tubulin is pulled, generating a mechanical force which breaks the microtubule [19], [31].

Katanin, spastin and fidgetin control microtubule flux during mitosis in *Drosophila melanogaster* [20], and recently, *Drosophila* katanin has also been shown to depolymerise microtubules and to regulate the interactions of microtubule plus ends with the cell cortex and to negatively regulate cell migration [32], [33]. Mammalian katanin is crucial for the redistribution of γ-tubulin at mitosis [34] while in *Chlamydomonas*, katanin is required for severing the flagellum from the basal bodies before mitosis [35] and in *Arabidopsis thaliana*, katanin was recently shown to be important for cell division [36]. Further, katanin protein, but not its microtubule severing activity is required for meiotic spindle formation in *Caenorhabditis elegans* [37], [38]. Spastin localises to centrosomes and spindle poles in dividing cells [39], and to the distal ends of axons, where it regulates axon branching [40]; mutations in the spastin gene, *SPG4,* account for 40% of cases of autosomal dominant Hereditary Spastic Paraplegia (HSP), which is characterised by the retrograde degeneration of corticospinal tracts (the longest axons), leading to progressive weakness and spastic paralysis of the lower limbs [41]. Additionally, spastin functions in the endosomal system in human cells, where two spastin isoforms are expressed. The 68 kDa isoform regulates endoplasmic reticulum (ER) to Golgi trafficking [42], and may be involved in ER shaping via interactions with ER membrane protein, receptor expression enhancing protein 1 (REEP1), and atlastin [43]. The shorter 60 kDa isoform localises to endosomes and is recruited to the midbody via an interaction with the endosomal sorting complex required for transport (ESCRT)-III machinery, where it seems to be required for severing microtubules to accomplish abscission [42], [44], [45]. Spastin knockdown has also been reported to result in the disorganization of central spindle microtubules and defective delivery of FIP3-containing endosomes to the intracellular bridge [46].

Since the trypanosome cytoskeleton needs to be bisected and remodelled during cell division, we reasoned that microtubule severing might play a major role in this process. Previously, the existence of homologues of the katanin p80 regulatory subunit (KAT80) and p60 catalytic subunit (KAT60a, KAT60b and KAT60c), spastin (SPA) and fidgetin (FID) in *T. brucei* was reported [47]. RNA interference (RNAi) studies of these proteins in procyclic trypanosomes, however, only yielded a discernible phenotype for KAT80; depletion of KAT80 slowed proliferation and was accompanied by the appearance of multi-nucleate cells and zoids (anucleate cells with a single kinetoplast), suggesting a possible role for KAT80 in cytokinesis [47]. The same study also investigated the subcellular localisation of these proteins in procyclic *T. brucei* using ectopically expressed tagged proteins. KAT60c could not be localised, but KAT80:GFP and KAT60a:GFP displayed a diffuse cytoplasmic localisation, and ectopic expression of KAT80:GFP disrupted cell division. Tagged KAT60b proteins localised to the flagellum and their expression reduced flagellar length by 20%. FID:GFP, GFP:SPA and SPA:GFP localised to the nucleus; FID:GFP had a punctate distribution during much of the cell cycle but localised to the spindle during mitosis while the tagged spastin proteins formed puncta around the trypanosome nucleolus throughout the cell cycle and their expression led to abnormalities in nucleus morphology [47].

Given the functional variability of several cell cycle and cytokinesis regulators between trypanosome life cycle stages [5], [14], and differences in the molecular composition of the cytoskeleton [48]–[50], we sought to investigate the function of the katanins, spastin and fidgetin in bloodstream stage *T. brucei*. While RNAi of fidgetin did not yield any discernible phenotype, depletion of the katanin and spastin proteins revealed that they play essential, non-redundant and discrete functions in cytokinesis in bloodstream trypanosomes.

## Results

To investigate the roles of katanins, spastin and fidgetin in bloodstream *T. brucei*, two or three independent RNAi cell lines were generated for each microtubule severing protein. Equivalent procyclic RNAi cell lines were also generated for comparative purposes. Following the induction of RNAi by the addition of tetracycline to the growth medium, the efficiency of the RNAi was assessed either by performing quantitative PCR to determine mRNA knockdown, or by replacing one allele of the gene in question with a tagged version, allowing determination of protein knockdown via Western blotting. The effects of microtubule severing protein depletion on proliferation and cell cycle progression were then monitored. Trypanosomes are easily classified into different cell cycle stages by staining the nucleus and mitochondrial genome (the kinetoplast). In early G_1_ phase, cells have one nucleus and one kinetoplast (1N1K). Kinetoplast S phase starts before nuclear S phase and is also substantially shorter, resulting in cells with a 1N2K configuration in early G_2_ phase. During mitosis, the nucleus divides to generate a 2N2K cell, which then undergoes cytokinesis to give rise to two daughter 1N1K cells. RNAi analysis revealed no discernible phenotype for fidgetin in either life cycle stage (data not shown), and hence this protein will not be considered further here. RNAi of the KAT60 proteins or spastin in procyclic *T. brucei* also did not yield any discernible phenotype (data not shown), in line with previous observations [47].

### KAT80 is required for normal proliferation and cell division in procyclic *T. brucei*

Depletion of KAT80 in procyclic *T. brucei* indicated that it is essential for normal *in vitro* growth (Fig. S1A and S1B), confirming previous observations [47]. Following KAT80 depletion, DAPI staining showed that 2N1K and 0N1K (zoid) cells slowly accumulated in equal numbers over the first 5 days of induction (Fig. S1C), suggesting that these abnormal cells were the progeny of a 2N2K cell, which had divided aberrantly. The zoid population continued to increase, comprising about 20% of the total population by 7 days post-induction (Fig. S1C) and flow cytometry profiles of propidium iodide-stained cells showed a peak of <1C DNA content appearing from 3 days post-induction, consistent with the zoid population observed by DAPI staining (Fig. S1D). Taken together, these data are consistent with KAT80 being important for accurate positioning of the cleavage furrow during cytokinesis in procyclic *T. brucei*.

### KAT80 is essential for proliferation and cleavage furrow ingression in bloodstream *T. brucei*

**Figure 1:**
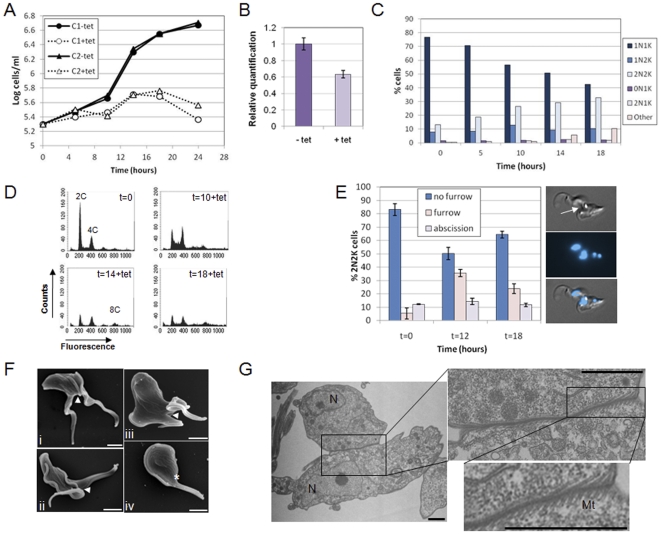
KAT80 is essential for cytokinesis in bloodstream stage *T. brucei*. A. Cumulative growth curves of two independent bloodstream *KAT80* RNAi clones (C1: clone 1; C2: clone 2) cultured in the presence or absence of tetracycline (tet). B–G show data for clone 1; data for clone 2 is comparable (not shown). B. Real time PCR analysis showing downregulation of *KAT80* transcript 14 hours post-induction with tetracycline (tet). Error bars represent standard deviations. C. DAPI staining. The nucleus (N) and kinetoplast (K) configurations of >200 cells per time point following RNAi induction are shown. ‘Other’ comprises mainly multinucleate and/or multi-kinetoplast cells. D. DNA content analysis. The fluorescence of 10,000 propidium iodide-stained cells was analysed by flow cytometry at the time points (in hours) indicated. The ploidies of peaks are given. E. Cytokinesis stage analysis. 2N2K cells (*n*>100 cells per time point) were analysed for their cytokinesis stage. Error bars represent standard deviations of 3 biological replicates. Example image of 2N2K furrowing cell shown on right. Top panel: DIC image; middle panel: DAPI image; bottom panel: merge. Arrow indicates the furrow. F. SEM analysis of induced *KAT80* RNAi cells (t = 12.5 hours). i-ii: furrowing cells; iii: furrowing cell with rounded posterior end; iv: cell with posterior end folded back towards the anterior. Arrowheads point to furrows while the asterisk indicates the posterior end in (iv). Scale bars: 2 µm. G. TEM analysis of a furrowing cell following induction (t = 12.5 hours) of *KAT80* RNAi, enlarged as indicated to visualise subpellicular microtubules (Mt). N: nucleus. Scale bars: 1 µm.

RNAi of *KAT80* in bloodstream trypanosomes resulted in a more dramatic phenotype than in procyclic parasites. A rapid decrease in growth rate followed by a growth arrest was observed within 24 hours of RNAi induction (Fig. 1A), despite only a rather modest reduction (∼40% at 14 hours post-induction) in *KAT80* mRNA (Fig. 1B). DAPI staining revealed that the proportion of post-mitotic (2N2K) cells in the population increased substantially (from 13.2% to 26.4%) over the first 10 hours of induction, rising to 32.8% at 18 hours post-induction (Fig. 1C). Unlike KAT80 depletion in procyclic *T. brucei*, few 0N1K and 2N1K cells were observed, but other abnormal cell types (mainly multi-nucleate and/or multi-kinetoplast cells) started to appear in small numbers at 14 hours post-induction and constituted 10% of the population at 18 hours post-induction. Flow cytometry data were consistent with the DAPI data, showing a relative increase in cells with a 4C DNA content 10 hours post-induction and the appearance of an 8C peak from 14 hours post-induction (Fig. 1D). The increase in 2N2K cells and subsequent appearance of multinucleate cells indicated that cytokinesis was delayed, since cells that do not divide in a timely fashion are known to re-replicate their organelles [5]. To determine whether the 2N2K cells were arrested at a particular stage of cytokinesis, 2N2K cells were scored according to how far cytokinesis had progressed (Fig. 1E). At t = 0, the majority of 2N2K cells were in early cytokinesis, since only about 15% of cells displayed a visible cleavage furrow, or had reached abscission, the final stage of cytokinesis. At later time points, there was a significant increase (one way ANOVA and Dunnett's post-hoc test; F = 55.30, df = 8, p = 0.00) in furrowing 2N2K cells (see example cell image in Fig. 1E) with a concomitant decrease in non-furrowing cells (Fig. 1E). Thus, depletion of KAT80 in bloodstream form cells appears to delay furrow ingression during cytokinesis. Scanning electron microscopy (SEM) was used to visualise *KAT80* RNAi cells in more detail (Fig. 1F). In addition to cells undergoing furrow ingression (Fig. 1F, images i -iii) being clearly visible following RNAi induction, defects in posterior end morphology such as rounding or broadening were observed in some cells (Fig. 1F, image iii) and in a few cells, the posterior end was unusually folded back towards the anterior of the cell (Fig. 1F, image iv). Furrows were further examined by transmission electron microscopy (TEM) (Fig. 1G), which showed microtubules arranged in a herring bone pattern at the furrow either side of the undivided cell membrane. No defects in microtubule spacing or membrane association were observed at the furrows (Fig. 1G) or elsewhere in the cell (Fig. S2), arguing that, in addition to a possible effect on microtubules at the posterior end of the cell, the depletion of KAT80 specifically affects microtubule cleavage and membrane remodelling at the furrow, but does not cause global defects in microtubule spacing or membrane attachment.

### KAT60a, KAT60b and KAT60c are essential for proliferation and are required for early events during cytokinesis in bloodstream trypanosomes

**Figure 2:**
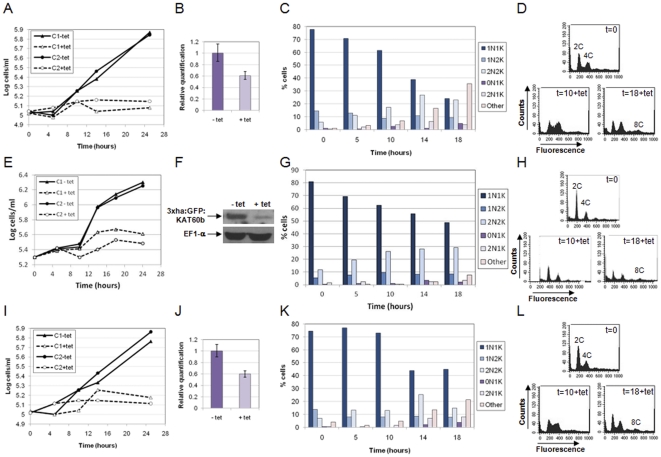
KAT60a, KAT60b and KAT60c are each essential for cytokinesis in bloodstream stage *T. brucei*. Panels A–D: KAT60a; panels E–H: KAT60b; panels I–L: KAT60c. A, E, I: cumulative growth curves of two independent bloodstream RNAi clones (C1: clone 1; C2: clone 2) cultured in the presence or absence of tetracycline (tet). B,F,J: real time PCR (KAT60a and KAT60c) or Western analysis (KAT60b), at 8 hours post-induction showing downregulation of the relevant transcript or protein (data presented for clone 1 in each case). Error bars represent standard deviations. For *KAT60b* RNAi cell lines (F), one allele of *KAT60b* was replaced with 3xha:GFP:KAT60b to allow visualisation of KAT60b downregulation by Western blotting with anti-HA antibody. The membrane was also probed with anti-elongation factor 1 alpha (EF1-α) antisera as a loading control. C, G, K: DAPI staining. The nucleus (N) and kinetoplast (K) configurations of >200 cells per time point following RNAi induction of clone 1 are shown. ‘Other’ comprises mainly multinucleate and/or multi-kinetoplast cells. D, H, L: DNA content analysis. The fluorescence of 10,000 propidium iodide-stained cells was analysed by flow cytometry in the FL2-A channel at the time points (in hours) indicated for *KAT60a*, *KAT60b* and *KAT60c* RNAi cell lines. The ploidies of peaks are given. Data for clone 2 are comparable (not shown).

Depletion of any of the KAT60 proteins resulted in a growth arrest within 24 hours in bloodstream stage *T. brucei* (Fig. 2A, 2B, 2E, 2F, 2I, and 2J), indicating that these proteins are essential for proliferation and are non-redundant. As specific antibodies to the trypanosome katanins are not yet available, tagged copies of the katanin genes were introduced at the respective endogenous loci in the RNAi cell lines to allow monitoring of protein knockdown following RNAi induction. However, only tagged KAT60b protein could be reliably detected by Western blot, revealing a substantial depletion of KAT60b at 8 hours post-induction (Fig. 2F). Since tagged KAT60a and KAT60c could not be detected, presumably due to very low levels of expression, real time PCR was used to show that *KAT60a* and *KAT60c* transcripts were downregulated to ∼60% at 8 hours post-induction (Fig. 2B, 2J).

In each case, depletion of KAT60a, KAT60b or KAT60c resulted in an increase in 2N2K cells, followed by the appearance at later time points of substantial numbers of multi-nucleate/multi-kinetoplast cells (Fig. 2C, 2G, and 2K), indicating defects in cytokinesis. Small numbers of 0N1K (zoid) and 2N1K cells were also present at later time points, and are also characteristic of defects in cytokinesis. Flow cytometry data were consistent with the DAPI data, showing an increase in the 4C peak relative to the 2C peak and the appearance of cells with >4C DNA content over time (Fig. 2D, 2H, and 2L). The kinetics of the cell cycle progression defects varied for the different KAT60 proteins. For example, depletion of KAT60b resulted in 2N2K cell numbers gradually increasing over the time course of the experiment with multinucleate cells only appearing later at 14–18 hours post-induction (Fig. 2G), while depletion of KAT60a or KAT60c saw 2N2K cell numbers peaking at ∼14 hours post-induction, at which time there were also significant numbers of multinucleate cells present (Fig. 2C and 2K). The differences between the KAT60 proteins may reflect subtle differences in their function.

**Figure 3:**
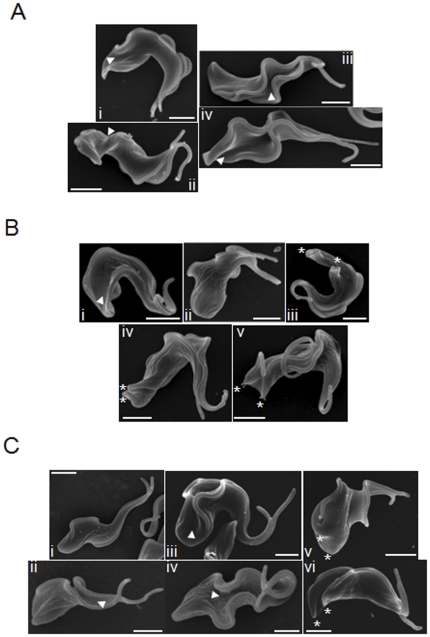
Depletion of KAT60a, KAT60b or KAT60c delays cytokinesis at an early stage in bloodstream trypanosomes. SEM images of RNAi cells (A: KAT60a; B: KAT60b; C: KAT60c) with 2 full length flagella. Cells were induced with tetracycline for 12.5 hours. Arrowheads indicate cleavage folds. Scale bars: 2 µm.

Examination of the 2N2K cell populations that accumulated post-RNAi induction for all three KAT60 proteins revealed only minor changes in the proportion of cells at different stages of cytokinesis (no furrow, furrowing and abscission) over time (Fig. S3), with around 80% cells having no visible furrow. However, examination of *KAT60a*, *KAT60b* and *KAT60c* RNAi cells at higher magnification by SEM revealed qualitative differences between them. Examination of cells with two full length flagella (2F), likely to correspond to the 2N2K cells visualised by DAPI staining [10], at 12 hours post-induction, revealed that following KAT60a depletion, in many of these cells without a furrow, a cleavage fold had formed along the longitudinal axis of the cell (Fig. 3A, images i-iv), although the two daughter cell bodies had not been physically separated at any point along their length. This fold was not easily visible by light microscopy, and thus 2N2K cells classified as having ‘no furrow’ by DAPI staining are likely to have included cells with and without cleavage folds. Fold formation likely represents the early stages of furrow ingression, characterised by membrane ingression and (presumably) remodelling of the underlying microtubules. The apparent abundance of 2F cells with cleavage folds following KAT60a depletion may suggest a particular importance for KAT60a in the final cleavage of the cytoskeleton to separate the daughter cell bodies. In contrast, following KAT60b depletion, 2F ‘no furrow’ cells, in general, did not show such a pronounced cleavage fold (Fig. 3B; compare Fig. 3B, image i with Fig. 3A, images iii and iv), while defects in posterior end morphology eg rounding were evident in some cells (Fig. 3B, image ii). Additionally, despite the absence of cleavage furrow ingression from the anterior end of the cell, two daughter cell bodies were defined, but not separated at the posterior end of some cells (Fig. 3B, images iii–v). This may indicate that KAT60b is important for microtubule and membrane remodelling at the anterior of the cell, and following its depletion, remodelling occurs preferentially at the posterior end. *KAT60c* RNAi 2F ‘no furrow’ cells showed a variety of morphologies, including no membrane invagination or cleavage fold formation (Fig. 3C, image i), substantial cleavage fold formation (Fig. 3C, images ii–iv), and definition of the two daughter cell posterior ends in the absence of cleavage at the anterior end (Fig. 3C, images v–vi), which may suggest that KAT60c plays a role in cytoskeleton/membrane remodelling throughout cytokinesis.

### Spastin is required for abscission in bloodstream stage *T. brucei*

**Figure 4:**
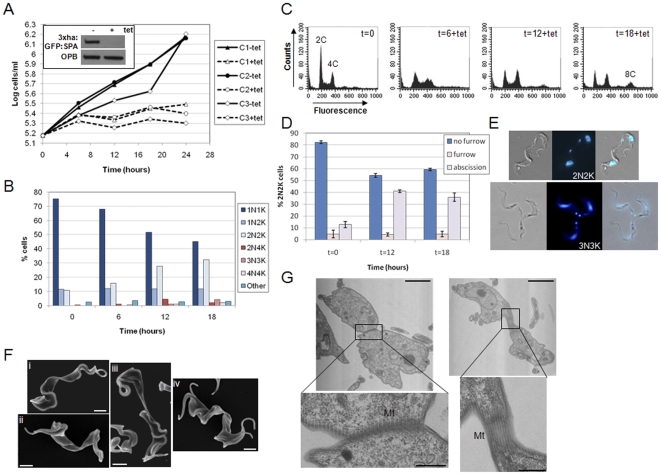
SPA is essential for abscission in bloodstream stage *T. brucei*. A . Cumulative growth curves of three independent bloodstream *SPA* RNAi clones (C1: clone 1; C2: clone 2; C3: clone 3) cultured in the presence or absence of tetracycline (tet). Inset: Western blotting showing downregulation of SPA expression in clone 3 following RNAi induction. One allele of *SPA* in the RNAi cell line was replaced with an epitope tagged copy of the gene (*3xha:GFP:SPA*). Whole cell lysates (12 hours post-induction with tetracycline (tet)) were analysed by Western blotting with anti-HA antibody (top panel). Lysates were also blotted with anti-oligopeptidase B (OPB) antibody as a loading control (lower panel). B-G show data for clone 3. B. DAPI staining. The nucleus (N) and kinetoplast (K) configurations of >200 cells per time point following RNAi induction are shown. C. DNA content analysis. The fluorescence of 10,000 propidium iodide-stained cells was analysed by flow cytometry at the time points (in hours) indicated. The ploidies of peaks are given. D. Cytokinesis stage analysis. 2N2K cells (*n*>100 per time point) were analysed for their cytokinesis stage. Error bars represent standard deviations of 3 biological replicates. E. Example images of 2N2K and 3N3K cells in abscission. Left panels: DIC image; middle panels: DAPI image; right panels: merge. F. SEM analysis of induced RNAi cells (t = 12.5 hours) in abscission. Scale bars: 2 µm. G. TEM analysis of *SPA* RNAi cells in abscission following induction with tetracycline (t = 12.5 hours), enlarged as indicated to visualise subpellicular microtubules (Mt) present at the contact region between the two daughter cell bodies. Scale bars: 2 µm (upper panels); 500 nm (lower panels).

Induction of *SPA* RNAi resulted in a rapid growth arrest, and depletion of SPA was confirmed by Western blotting, showing that spastin was essentially undetectable by 12 hours post-induction (Fig. 4A). Similar to the downregulation of KAT80 and KAT60, depletion of SPA resulted in a steady increase in 2N2K cells over time as observed by DAPI staining, reaching 32% at 18 hours post-induction (Fig. 4B) although, compared to depletion of the KAT60a and KAT60c, relatively few multinucleate cells were observed. Flow cytometry data was consistent with the DAPI data (Fig. 4C). Examination of the 2N2K cells revealed a highly significant increase in the proportion of 2N2K cells undergoing abscission from 13% to 41% at 12 hours post-induction ((one way ANOVA and Dunnett's post-hoc test; F = 106.80, df = 8, p = 0.00); Fig. 4D, 4E). Amongst the multinucleate cells, cells that had not yet completed abscission were observed re-replicating their DNA, reinitiating cytokinesis and again being blocked at abscission (see 3N3K cell in Fig. 4E). SEM analysis supported the DAPI data, showing cells with 2 full length flagella undergoing abscission (Fig. 4F, image i) as well as cells with multiple flagella apparently blocked in abscission (Fig. 4F, image ii-iv). SEM also revealed a few cells undergoing abscission to reveal two different posterior ends, a flat end and a pointed end (Fig. 4, image iii). TEM analysis of *SPA* RNAi cells undergoing abscission demonstrated that the two cell bodies remained joined by the plasma membrane and subpellicular microtubules, indicating that final microtubule severing and membrane remodelling had yet to occur (Fig. 4G). No defects in microtubule spacing or membrane association were observed (Fig. S4). Taken together, these data provide strong evidence for SPA playing an essential role in abscission, and we propose that spastin is important for the final severing steps required to separate the daughter cell subpellicular microtubule cytoskeletons.

### **Discussion**

One of the most remarkable, yet poorly understood, features of trypanosome cytokinesis is the cleavage and remodelling of the subpellicular microtubule cytoskeleton. During cytokinesis, the plasma membrane must be split to generate two daughter cells, and the underlying microtubule cytoskeleton divided. As the cleavage furrow ingresses along the longitudinal axis of the cell, the membrane invaginates and crosslinks between the microtubules situated either side of the division plane are presumably broken. The trypanosome has a vermiform shape, conferred by a corset of parallel microtubules running longitudinally. The wider the cell is at any point along its length, the greater the number of microtubules that are present at that point, and the longest microtubules are found in the middle of the cell. Thus, furrow ingression will require microtubules to be severed and shortened or re-formed on either side of the cleavage plane, to maintain the vermiform shape in the daughter cells. Indeed, an increase in new, short, tyrosinated (see below) microtubules is observed in the middle of post-mitotic procyclic cells [51], and microtubule severing may aid the synthesis of new microtubules by increasing the pool of free tubulin subunits. Crosslinks between the microtubules and the plasma membrane are also likely to be remodelled as the furrow ingresses.

Our functional analysis of microtubule severing protein homologues reveals that KAT80, KAT60a, KAT60b, KAT60c and SPA are all essential (and therefore non-redundant) for cytokinesis in bloodstream trypanosomes and provides the first clues as to how division of the cytoskeleton is accomplished. Depletion of the katanin and spastin proteins each resulted in the accumulation of 2N2K cells but interfered with cytokinesis at different stages, suggesting that the coordinated action of several microtubule severing enzymes is required to divide the trypanosome cytoskeleton. The KAT60 proteins appear to be important early on in cytokinesis, since the majority of 2N2K cells that accumulated following their depletion did not display cleavage furrows. Our data suggest that KAT60a may be important for conversion of the cleavage cleft to a furrow to separate the two daughter cell bodies. KAT60b on the other hand appears to be important for cleavage of the cytoskeleton at the anterior of the cell, since following its depletion, cells were observed with defined (although not separated) posterior daughter cell ends despite the lack of furrow ingression from the anterior. KAT60c is perhaps important at multiple stages of cytokinesis as no dominant morphological phenotype was observed amongst the 2N2K/2F cell population. Depletion of KAT80 and SPA affected later stages of cytokinesis, specifically inhibiting furrow ingression and abscission, respectively, as evidenced by the increased proportion of 2N2K cells with a partially ingressed cleavage furrow, or just joined at their posterior tips. One possible caveat in this interpretation of our data is that it is possible that the degree of protein knockdown induced by the RNAi for each of these severing proteins influences the stage of cytokinesis affected. For example, KAT80 and/or spastin could be required throughout cytokinesis, but in lower amounts during the early stages, and protein remaining after RNAi induction may be sufficient for cytokinesis until cells reach furrow ingression/abscission, or alternatively, there could be redundancy of function between the microtubule severing enzymes during some stages of cytokinesis but not others. However, since the phenotypes between independent RNAi cell lines for any given protein were highly consistent and very distinctive phenotypes were observed for the different severing proteins, we favour a model where these severing proteins play distinct roles at different stages of cytokinesis.

It is possible that each protein has different microtubule cleavage specificities, for example due to differences in microtubule post-translational modifications and/or differential interactions with microtubule-binding proteins, which vary through cytokinesis. Indeed, it has been proposed that *Tetrahymena* Kat1 preferentially severs older post-translationally modified microtubules, which would account for its specificity for only a subset of microtubules [52] and acetylation and polyglutamylation of tubulin have been shown to enhance katanin and spastin-mediated microtubule severing, respectively in mammalian cells [29], [30]. Further, microtubule-associated proteins XMAP230 in *Xenopus*, Tau and MAP2 in mammalian cells and MAP65-1c in tobacco have been shown to reduce katanin-mediated severing of microtubules [26]–[28], most likely by blocking katanin binding sites on the microtubules. *T. brucei* microtubules are known to be modified by acetylation, tyrosination and polyglutamylation [53]. Acetylation of lysine 40 of α-tubulin is a stable modification found on all microtubules - the stable subpellicular and axonemal microtubules as well as the transient spindle microtubules - that is incorporated during microtubule polymerisation [54]. Recently, depletion of the GTPase ARL2 was shown to lead to reduced levels of acetylated α-tubulin and defects in cytokinesis [16]; whether the two observations are directly linked, perhaps by lower levels of microtubule acetylation modulating microtubule severing, remains to be seen. Glutamylation and tyrosination have been detected on both α and β tubulin [11], [55]. Microtubules become detyrosinated over time, and in procyclic cells, there are cell cycle differences in the tyrosination status of microtubules, with cells in early stages of the cell cycle displaying the most tyrosinated microtubules, and cells around mitosis displaying the least [51]. However, little is currently known about other potential post-translational modifications of microtubules, such as cell cycle dependent phosphorylation, which could also play a role in regulating microtubule severing. A number of trypanosome cytoskeleton or microtubule-associated proteins (MAPs) [48]–[50], [56]–[62] have been identified, and in the case of CAP15 and CAP17, shown to increase the stability of microtubules [48]. Depletion of the MAPs CAP5.5 and WCB leads to aberrant cytokinesis [50], [56] due to resultant defects in cytoskeleton stability, structure or polarity and their downstream consequences; whether decreased cytoskeleton stability results directly from the loss of the MAP or indirectly due to the loss of the MAP increasing the availability of sites on the microtubules that can be utilised by microtubule severing enzymes is not clear at present.

The apparent role of KAT80 in promoting furrow ingression is intriguing given that p80 katanins do not have any microtubule severing activity and are involved in microtubule severing only via their interactions with p60 katanin subunits. However, to date, despite repeated attempts, we have been unable to detect an interaction between KAT80 and KAT60b or KAT60c either in non-synchronised or hydroxyurea-synchronised cytokinesis cell extracts (Fig. S5 and data not shown). We cannot rule out that KAT80 interacts with KAT60b or KAT60c, perhaps very transiently during cytokinesis, or that it interacts with KAT60a, as tagged KAT60a proteins remained below the detection limit of Western blotting, precluding analysis of their interactions with KAT80. The function of the KAT60 proteins early in cytokinesis may occur independently of KAT80, which appears to be required later during furrow ingression, since katanin p60 subunits are known to be enzymatically active and functional in the absence of p80 katanin [24]. However, interaction of the KAT60 proteins with KAT80 later in cytokinesis could alter their function, since binding of the *C. elegans* p80 subunit, MEI-2, to p60 katanin (MEI-1) was shown to dramatically change its microtubule binding properties [38].

A role for katanins in cytokinesis has not been widely reported. Studies of KAT80 and KAT60a in the related kinetoplastid, *Leishmania major*, suggest they may play a role in cytokinesis, since they relocalise to the cleavage furrow during cytokinesis [47], but to date, the only other evidence linking katanins with cytokinesis comes from studies of the LAPSER1 candidate tumour suppressor gene for prostate cancer in a rat cell line [63], [64]. Overexpression of LAPSER1 results in defects in central spindle formation during cytokinesis that lead to the formation of binucleated cells. LAPSER1 binds to p80 katanin, inhibiting p60 katanin microtubule severing activity and p80 translocates in a LAPSER1-dependent manner from the centrosomes to the midbody during cytokinesis [63], and thus, the observed defects following LAPSER1 overexpression may be mediated, at least in part, through the inhibition of katanin. However, trypanosome mitosis and cytokinesis are highly divergent compared to mammalian cell division. For example, mitosis is closed, DNA does not condense, trypanosomes do not have centrosomes and as discussed earlier, the mechanics of cytokinesis are very different [5], . Additionally, while trypanosome daughter cells may remain joined at their posterior ends for some time prior to abscission, a midbody-like structure has not been described. Thus, while it is unlikely that trypanosome katanins operate in an analogous manner to mammalian katanin, it appears that that *T. brucei* has exploited the microtubule severing activities of katanins to achieve efficient cytoskeleton cleavage during cell division.

Katanins play a variety of other roles in other organisms, being required for regulating the length of meiotic and mitotic spindles [37], [65], [66], assembly and disassembly of flagella and cilia [35], [52], [67], and neuronal cell morphogenesis [40], [68]–[70]. No role was noted for any of the katanins in mitosis or flagellar physiology in bloodstream trypanosomes in this study. An accumulation of 2N2K cells following RNAi of the katanins and lack of obvious defects in nucleus morphology, and the absence of spindles by TEM, suggests mitosis was unaffected by their depletion, although without conditional katanin knockout cell lines, a role for these proteins in mitosis cannot be completely excluded. It is also possible that the katanins are required for mitosis, but, unlike for their cytokinesis functions, there is a degree of redundancy between the proteins. However, our data suggest that mitosis in bloodstream *T. brucei* may not be regulated by the action of katanins. This is not without precedence, since katanins are not present in the mitotic spindle of *Caenorhabditis elegans* [71], [72]. It is also known that the kinesin, TbKif13-1, regulates mitotic spindle length in *T. brucei*; its depletion results in the formation of abnormally long spindles during mitosis, while its overexpression leads to an early mitotic block and lack of spindle formation [73], [74], and two other kinesin-like proteins, KINA and KINB also appear to be important for the establishment of the mitotic spindle [75], and hence, katanins may not be required for mitotic spindle formation. Detailed analyses of flagella were not performed during this study, but no defects in flagellar motility were observed, and all flagella cross-sections viewed by TEM for *KAT80* and *SPA* RNAi cell lines appeared normal (Figs. S2, S4 and data not shown), suggesting that depletion of the katanins was unlikely to have caused gross structural defects, such as the loss of the central pair microtubules, which occurs in flagella following mutation of p80 katanin in *Chlamydomonas* [67] or in cilia after deletion of p60 katanin in *Tetrahymena* [52].

Our data also identify a role for spastin in abscission in bloodstream *T. brucei*, consistent with the known function of the human spastin 60 kDa isoform at the midbody restructuring microtubules in the intercellular bridge to help bring about abscission [42], [45]. Despite being unable to localise spastin in this life cycle stage, its RNAi phenotype suggests that it is required for the final severing of microtubules at the posterior ends of the daughter cells. Spastin may play a different role in procyclic *T. brucei* since ectopically expressed GFP-tagged spastin localised to the nucleus and affected nuclear morphology, possibly suggesting a role in mitosis in this life cycle stage [47], although mislocalisation due to ectopic expression or the fluorescent tag cannot completely be ruled out. However, life cycle-specific functions for cell cycle regulators are common in *T. brucei*, and particularly in this case, may reflect differences in the composition of the cytoskeleton, based on differential expression of MAPs [48]–[50], and, as discussed above, potential differences in post-translational modifications, which may affect the binding of microtubule severing proteins to the cytoskeletal microtubules [27]–[29].

In summary, cytokinesis requires a family of katanins and spastin in bloodstream *T. brucei*, while mitosis may not. While the presence of multiple p60 katanin proteins is not without precedence (*Drosophila*, for example, has three: CG10229, which is most similar to human p60 and is essential for microtubule dynamics during mitosis [20], CG1193, which is involved in dendrite severing in sensory neurons during metamorphosis [76] and CG10793, which has not yet been characterised), the expression of three KAT60 proteins that play essential and non-redundant roles in cytokinesis likely reflects the challenges inherent in cleaving the stable microtubule cytoskeleton in *T. brucei*. It is interesting that KAT60c (and also spastin) are not present in *L. major,* and that in promastigote *Leishmania*, while the subcellular localisation of KAT60a suggests it may play a role in cytokinesis, KAT60b appeared to be involved in flagellar length regulation [47]. Presumably these differences reflect differences in the cell biology and structure of these two parasites. Depletion of the microtubule severing proteins in *T. brucei* did not appear to cause widespread disruption to the trypanosome cytoskeleton although more detailed electron microscopy investigations of the trypanosome cytoskeleton following depletion of katanins and spastin will be required to rule out subtle defects. Evidence for KAT80 regulating the KAT60 proteins, as has been found to be the case for all other p80 katanins studied to date, is still lacking in *T. brucei*, and the role of fidgetin remains enigmatic. To date, we have unfortunately been unable to detect any tagged katanin or spastin protein in bloodstream form trypanosomes via immunofluorescence, presumably due to them being present at very low levels in the cell, and thus have been unable to determine their subcellular localisation, which would shed further light on their functions. Investigating the interactions between the different katanins, and between the microtubule severing enzymes and the microtubule cytoskeleton will be challenging due to their very low abundance, but should be a priority in the future, as should purifying these proteins to allow their biochemical characterisation. Pairwise knockdown of different katanins may provide further insight into their overlapping roles in cytokinesis, and a fidgetin gene knockout should allow an assessment of its essentiality.

## Materials and Methods

### Cloning of RNAi, endogenous tagging and inducible expression constructs

Gene products were amplified from *T. brucei brucei* strain EATRO 795 or Lister 427 genomic DNA using the polymerase chain reaction (PCR) with the oligonucleotides detailed in Table S1. PCR products were cloned into pSC-B (Stratagene), pGEMTeasy (Promega) or pCR2.1 TOPO (Invitrogen) and sequenced (by The Sequencing Service, University of Dundee) to check the fidelity of the PCR. Gene products were then subcloned into the appropriate destination vectors using the restriction sites incorporated into the primers (Table S1).

For RNAi, unique fragments of *KAT80, KAT60a, KAT60b, KAT60c, SPA* and *FID* (selected by the programme RNAit [77], to minimise the possibility of off-target effects) were subcloned into p2T7_ti_:*GFP* [78] in place of the *GFP* sequence, generating pGL1103 (*KAT80*), pGL1049 (*KAT60a*), pHG96 (*KAT60b*), pGL1101 (*KAT60c*), pHG99 (*SPA*) and pHG94 (*FID*).

Genes were also tagged at their 5̀ ends within their endogenous locus to retain the endogenous 3̀ UTR sequence and maintain native expression levels as closely as possible. The 5̀ end of the open reading frame (ORF) and a region encompassing the 5̀ untranslated region (UTR) just upstream of the start codon were amplified by PCR and subcloned via a three-way ligation into pEnT vectors [79] or their derivatives (pHG35 and pHG80) as described here. The endogenous tagging vectors generated were: pHG84 (*3xha:eGFP:KAT80* in pHG80), pHG38 (*ty:CFP:KAT60a* in pEnT6-PURO-CFP-TY), pHG142 (*3xha:eGFP:KAT60b* in pHG80), pHG47 (*myc:mCherry:KAT60c* in pHG35) and pHG111 (*3xha:eGFP:SPA* in pHG80). pHG35 was generated by amplifying *c-myc:mCherry* from p2686 [79] using PR7 and PR29 (Table S1) and cloning this sequence using Hind III/Xba I into pEnT6BLAST-CFP-TY in place of *ty:CFP*. pHG80 was constructed by digesting pEnT6BLAST-eGFP-TY with Hind III and Spe I to remove the TY epitope coding sequence and replacing it with annealed oligonucleotides (PR9 and PR10) encoding a 3xHA epitope tag. Expression of tagged proteins following transfection of the constructs into *T. brucei* (see below) was verified by Western blotting with appropriate antibodies against the tags. Due to KAT60 tagged proteins being barely detectable or undetectable, presumably due to low expression levels, inducible expression constructs were generated for KAT60a (KAT60a:ty; pHG139) and KAT60c (KAT60c:myc; pGL1502) in a pHD675 background [80]; KAT60a still could not be detected by Western blotting with anti-TY antibody following induction.

### Culturing and transfection of T. brucei and analysis of cell lines

Bloodstream and procyclic stage *T. brucei brucei* were cultured and transfected as described previously [4], [81]. For RNAi, the procyclic 427 pLew13 pLew29 and the bloodstream form 427 pLew13 pLew90 cell lines [82] were transfected with the appropriate Not I-linearised, purified RNAi plasmids (described above) and cloned by limiting dilution. Induction of RNAi was achieved by adding 1 µgml^-1^ tetracycline to the culture medium. To assess the level of specific mRNA downregulation following induction of RNAi, real time PCR analysis was performed on cDNA prepared from RNAi cell lines at a suitable time point following induction, as described previously [83]. The gene specific primers used are given in Table S1; control primers were for *GPI8* [83], [84]. To enable downregulation of the targeted protein to be monitored by Western blotting, RNAi cell lines were transfected with the appropriate endogenous tagging constructs (described above), linearised with *Xba* I. Lysates were analysed by Western blotting with antibodies against the epitope tag (see below); loading controls were performed by staining the membrane with Ponceau S, or by blotting with anti-oligopeptidase B (OPB) [85] or anti-elongation factor 1 alpha (EF1-α; clone CBP-KK1, Millipore) antisera. Following induction of RNAi cell lines, cell proliferation was monitored by counting cells at appropriate intervals using a Neubauer Improved haemocytometer and cell cycle progression was analysed via DAPI staining of methanol-fixed cells and flow cytometry of propidium iodide stained cells as described previously [4]. The cytokinesis stage of 2N2K cells was determined as previously described [86] and the results were analysed by one way ANOVA and a Dunnett's post-hoc test.

For analysis of subcellular localisation of katanins and spastin, and interactions between katanin proteins, *Xho* I-linearised endogenous tagging constructs were transfected into Lister 427 bloodstream stage cells. Where appropriate, *T. brucei* bloodstream stage cultures were synchronised using hydroxyurea as described previously [87] and the success of the synchronisation monitored by DAPI staining and flow cytometry (as above). Inducible expression constructs were *Not* I-linearised, transfected into the bloodstream stage cell line 427 pHD449 [80], and protein expression induced by adding 1 µgml^−1^ tetracycline to the culture medium. Expression of tagged proteins was assessed using Western blotting of cell lysates with anti-TY [88], anti-GFP (Santa-Cruz), anti-HA (Roche) and anti-myc (GenScript) antibodies. To analyse interactions between katanin proteins, 1–5×10^9^ bloodstream form cells expressing 3xha:GFP:KAT80 and a tagged copy of KAT60b or KAT60c were lysed in 1 ml ice cold lysis buffer (50 mM Tris-Cl pH 7.6, 150 mM NaCl, 10% glycerol, 0.1% NP40, 1 mM EDTA, 1 mM EGTA) containing protease inhibitors (234 µM leupeptin, 500 µM phenylmethanesulphonylfluoride (PMSF), 7.3 µM pepstatin A, 2.5 µM pefabloc, 250 µM 1,10 phenanthroline and 10 µM E-64d) and incubated for 15 minute on ice. Cell debris was removed by centrifuging at 100,000×*g* at 4°C for 45 minutes. The resulting supernatant was incubated with 100 µl anti-HA affinity matrix (Roche) for 2 hours at 4°C to immunoprecipitate 3xha:GFP:KAT80. Following 4 washes with 1 ml of wash buffer (50 mM Tris-Cl pH 7.6, 150 mM NaCl, 10% glycerol, 0.1% NP40), immunoprecipitated proteins were eluted by boiling in Laemmli buffer. Eluted proteins were analysed by SDS-PAGE followed by Western blotting probing with antibodies against the tags (as above).

### Electron microscopy

Specimens were prepared for electron microscopy by Laurence Tetley and Margaret Mullin, Integrated Microscopy Facility, University of Glasgow. For scanning electron microscopy (SEM) analyses, approximately 10^7^ bloodstream stage *T. brucei* cells were centrifuged at 1500×*g* for 5 minutes and washed once in Trypanosome Dilution Buffer (TDB,[87]) before being resuspended in 100 µl TDB and then fixed in 2.5% glutaraldehyde in 0.1M sodium cacodylate buffer (SC) for 1 hour. Cells were washed 3 times in 0.1M SC, before a droplet of concentrated cell suspension in 0.1M SC was placed onto a 10 mm diameter poly-L-lysine coated glass coverslip and cells allowed to settle onto the coverslip for 30 minutes. Following a brief rinse in 0.1M SC to remove unattached cells, coverslips were incubated in 1% osmium tetroxide in 0.05 M SC for 1 hour. Coverslips were then washed 3 times in dH_2_O before being incubated with 0.5% uranyl acetate in the dark for 1 hour. Uranyl acetate was removed by 2 washes with dH_2_O and cells were dehydrated in an alcohol series before being subjected to critical point drying for 1 hour 20 minutes. Specimens were then mounted on aluminium stubs using double sided copper tape and coated with gold/palladium in a Polaron SC515 sputter coater prior to examination using a JEOL 6400 scanning electron microscope operating at 6 kV. Images were captured using an ADDA3 digital capture system (Olympus/SIS, Germany).

For transmission electron microscopy (TEM), a pellet of approximately 10^7^ *T. brucei* bloodstream stage cells was fixed in 2.5% glutaraldehyde in 0.1M SC for 1 hour. Following 3 washes in 0.1M SC, the fixed pellet was incubated with 1% osmium tetroxide/0.05M SC for 1 hour, washed 3 times in dH_2_O and then incubated in 0.5% uranyl acetate in the dark for 1 hour. Following 2 rinses in dH_2_O, the cell pellet was dehydrated in an alcohol series, incubated in 3 changes of propylene oxide, before being incubated overnight in a 50∶50 mix of propylene oxide/TAAB araldite 502/512 resin. The propylene oxide was then allowed to evaporate to leave pure resin, which was changed twice before the pellet was embedded in fresh resin, which was allowed to polymerise at 60°C for 48 hours prior to cutting 60–70 nm thick sections and contrast staining with 2% uranyl acetate in methanol for 5 minutes and Reynolds' lead citrate for 5 minutes.

## Supporting Information

Figure S1 **KAT80 is important for normal proliferation and cytokinesis in procyclic** ***T. brucei*** **.** A. Cumulative growth curves of two independent procyclic *KAT80* RNAi clones. B. Western blotting showing downregulation of KAT80 expression following RNAi induction. One allele of *KAT80* was replaced with an epitope tagged copy of the gene (*3xha:GFP:KAT80*) in each of the *KAT80* RNAi cell lines in (A). Whole cell lysates (5 days post-induction with tetracycline (tet)) were analysed by Western blotting with anti-HA antibody (top panels). Portions of the Western membrane, stained with Ponceau after transfer (lower panels), are included as loading controls. C. DAPI staining. Following RNAi induction, cell cycle progression was monitored by DAPI staining at the time points indicated in days (d) (*n*>200). The nucleus (N) and kinetoplast (K) configurations of cells are given. ‘Others’ represents multinucleate/multikinetoplast cells. Data presented are for clone 1 and are representative of data for clone 2 (not shown). D. DNA content analysis. The fluorescence of 10,000 propidium iodide-stained cells was analysed by flow cytometry in the FL2-A channel at the time points (in days) indicated. The ploidies of the peaks are given.
(TIF)

Figure S2 **TEM analysis of microtubule spacing following induction of** ***KAT80*** **RNAi.** Transverse cross-sections of cells plus or minus induction with tetracycline (tet) for 12.5 hours are shown. Regions indicated by the black boxes are enlarged 3 fold to visualise the subpellicular microtubules. Scale bars: 1 µm.
(TIF)

Figure S3 **Cytokinesis stage analysis of 2N2K cells following depletion of KAT60a, KAT60b or KAT60c.** A–C show data for KAT60a, KAT60b and KAT60c, respectively. *n*>100 cells per time point. Error bars represent the standard deviation of 3 biological replicates.
(TIF)

Figure S4 **TEM analysis of microtubule spacing following induction of** ***SPA*** **RNAi.** Transverse cross-sections of cells plus or minus induction with tetracycline (tet) for 12.5 hours are shown. Regions indicated by the black boxes are enlarged 3 fold to visualise the subpellicular microtubules. Scale bars: 500 nm.
(TIF)

Figure S5 **3xha:GFP:KAT80 does not appear to interact with KAT60c:myc.** Co-immunoprecipitation analysis of interactions between 3xha:GFP:KAT80 and KAT60c:myc. Anti-HA antibody was used to immunoprecipitate 3xha:GFP:KAT80 from cell lysates co-expressing KAT60c:myc, as shown by Western blot analysis with anti-HA antibody (top panels) of input (I), flow through (FT), first and last wash (W1 and W4, respectively) and elution (E) fractions. The input, flow through and elution fractions were then blotted with an anti-myc antibody to detect KAT60c:myc.
(TIF)

Table S1 **Oligonucleotides used in this study.**
(DOC)
