# Correction: Cytokinesis in Bloodstream Stage *Trypanosoma brucei* Requires a Family of Katanins and Spastin

**DOI:** 10.1371/annotation/aa6cd97c-cfb5-4afe-aa25-f5ecfed07980

**Published:** 2012-03-07

**Authors:** Corinna Benz, Caroline Clucas, Jeremy C. Mottram, Tansy C. Hammarton

There was an error in the funding statement. The correct funding statement is: "This work was funded by the Medical Research Council (via a Career Development Fellowship (ref. G120/1001) and New Investigator Research Grant (ref. GO900239) to TCH), Research Councils UK (Academic Fellowship to TCH) and The Wellcome Trust (Project grant ref. 070231 to JCM and TCH, and Value In People funding ref. 084546/Z/07/Z to TCH for CB). The Wellcome Trust Centre for Molecular

Parasitology is supported by core funding from the Wellcome Trust

[085349/Z/08/Z]. The funders had no role in study design, data collection and analysis, decision to publish, or preparation of the manuscript." 

