# Supplementary figures and images for: Cytokinesis in Bloodstream Stage Trypanosoma brucei Requires a Family of Katanins and Spastin
Source: PLoS One. 2012 Jan 18;7(1):e30367. doi: 10.1371/journal.pone.0030367 (PMC3261199; doi:10.1371/journal.pone.0030367)

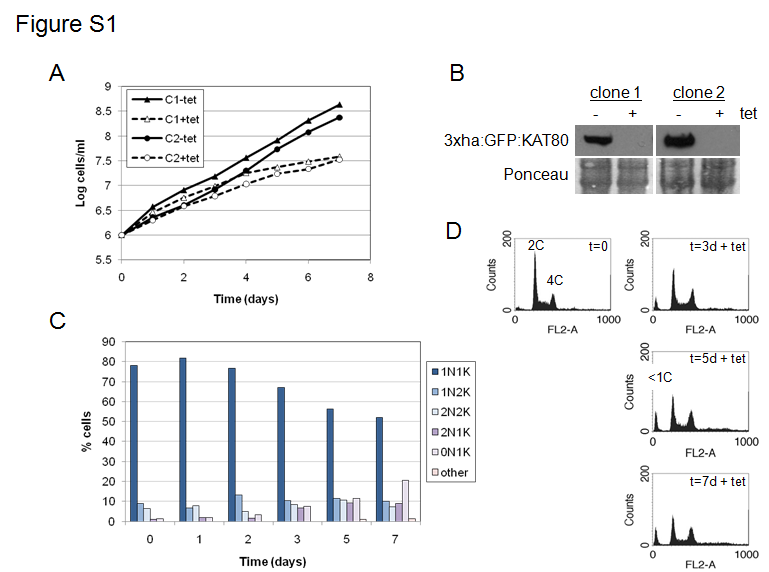

Supplement: Figure S1 — KAT80 is important for normal proliferation and cytokinesis in procyclic T. brucei . A. Cumulative growth curves of two independent procyclic KAT80 RNAi clones. B. Western blotting showing downregulation of KAT80 expression following RNAi induction. One allele of KAT80 was replaced with an epitope tagged copy of the gene (3xha:GFP:KAT80) in each of the KAT80 RNAi cell lines in (A). Whole cell lysates (5 days post-induction with tetracycline (tet)) were analysed by Western blotting with anti-HA antibody (top panels). Portions of the Western membrane, stained with Ponceau after transfer (lower panels), are included as loading controls. C. DAPI staining. Following RNAi induction, cell cycle progression was monitored by DAPI staining at the time points indicated in days (d) (n>200). The nucleus (N) and kinetoplast (K) configurations of cells are given. ‘Others’ represents multinucleate/multikinetoplast cells. Data presented are for clone 1 and are representative of data for clone 2 (not shown). D. DNA content analysis. The fluorescence of 10,000 propidium iodide-stained cells was analysed by flow cytometry in the FL2-A channel at the time points (in days) indicated. The ploidies of the peaks are given. (TIF) [file pone.0030367.s001.tif]

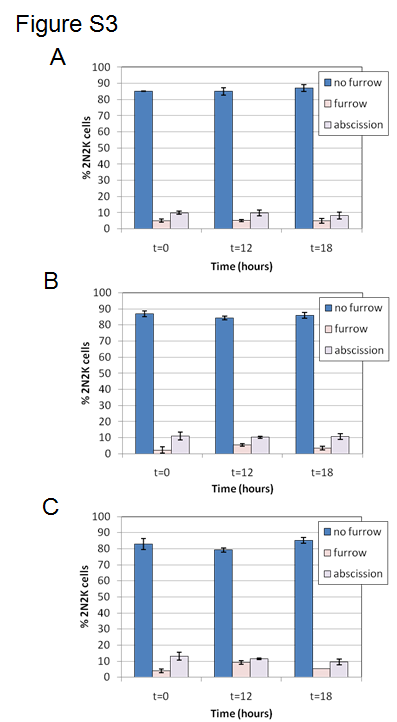

Supplement: Figure S3 — Cytokinesis stage analysis of 2N2K cells following depletion of KAT60a, KAT60b or KAT60c. A–C show data for KAT60a, KAT60b and KAT60c, respectively. n>100 cells per time point. Error bars represent the standard deviation of 3 biological replicates. (TIF) [file pone.0030367.s003.tif]

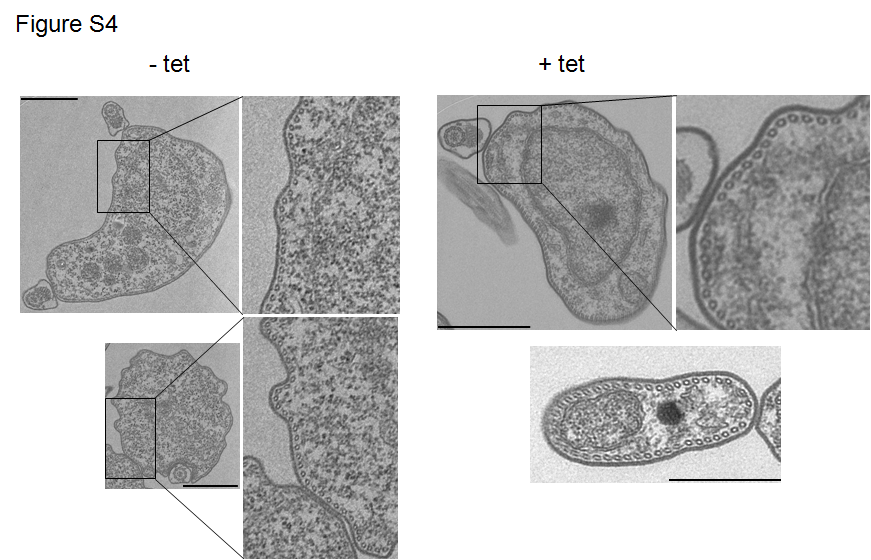

Supplement: Figure S4 — TEM analysis of microtubule spacing following induction of SPA RNAi. Transverse cross-sections of cells plus or minus induction with tetracycline (tet) for 12.5 hours are shown. Regions indicated by the black boxes are enlarged 3 fold to visualise the subpellicular microtubules. Scale bars: 500 nm. (TIF) [file pone.0030367.s004.tif]

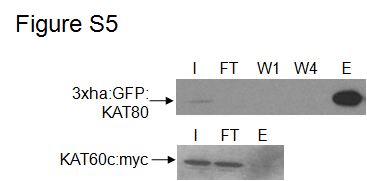

Supplement: Figure S5 — 3xha:GFP:KAT80 does not appear to interact with KAT60c:myc. Co-immunoprecipitation analysis of interactions between 3xha:GFP:KAT80 and KAT60c:myc. Anti-HA antibody was used to immunoprecipitate 3xha:GFP:KAT80 from cell lysates co-expressing KAT60c:myc, as shown by Western blot analysis with anti-HA antibody (top panels) of input (I), flow through (FT), first and last wash (W1 and W4, respectively) and elution (E) fractions. The input, flow through and elution fractions were then blotted with an anti-myc antibody to detect KAT60c:myc. (TIF) [file pone.0030367.s005.tif]
