# Supplementary material for: Cytokinesis in Bloodstream Stage Trypanosoma brucei Requires a Family of Katanins and Spastin
Source: PLoS One. 2012 Jan 18;7(1):e30367. doi: 10.1371/journal.pone.0030367 (PMC3261199; doi:10.1371/journal.pone.0030367)
Supplement: Table S1 — Oligonucleotides used in this study. (DOC) [file pone.0030367.s006.doc]

**Table S1. Oligonucleotides used in this study**

| **Gene** | **RNAi** | **Endogenous tagging** | **Ectopic expression** |
| --- | --- | --- | --- |
| *KAT80* | OL1592: GGGAAGCTTGTTTTCACCCAAAGAGATAC (Hind III) | PR15: CACATCTAGACCAGTTATCGATTCGATGAC (Xba I) |  |
|  | OL1593: GTACAGACATTTTAGCATTC | PR16: CCACCTCGAGCACAACCACTAACGCACC (Xho I) |  |
|  |  | PR17: CAATCTCGAGCAGGAAGACGCACACAGTG (Xho I) |  |
|  |  | PR18: GTGCGGATCCATTTTCGGTCTGTTCCTACTTTCG (Bam HI) |  |
| *KAT60a* | OL1513: GTCAGGATCCAGTTCAAACCGATTTGTGGC (Bam HI) | PR3: GAACTCTAGAAGCACATCTAATGACGTGC (Xba I) | PR1: GCACAAGCTTATGAGCACATCTAATGACG (Hind III) |
|  | OL1514: GGGAAGCTTCCCAACCAACACTAAGCGAT (Hind III) | PR4: CCTTCTCGAGCCTTATCGCCATACAGCAG (Xho I) | PR2: GAGGACGCGTTCAGTCAAGTGGATCCTGGTTAGTATGGACCTCAACATTTACCTCGAACTCC (Mlu I) |
|  |  | PR5: GGCACTCGAGGGTTAGGAGGAGGGAGTCTG (Xho I) |  |
|  |  | PR6: CACAGGATCCATCACTCCTTCTACACTTTGAGGG (Bam HI) |  |
| *KAT60b* | PR71: ATAGGATCCGCTCCAGCAGATAAAGGTGC (Bam HI) | PR140: TTCTAGAAGTAAAAAGGGTAACATTCGCC (Xba I) |  |
|  | PR72: TATCTCGAGCCAATGGAGGAGATGCCTTA (Xho I) | PR141: TAACTCGAGTGCGCAGTGCTTTGAGGTGC (Xho I) |  |
|  |  | PR138: TCTCGAGGTTCACTCCCATATTAGGTGTG (Xho I) |  |
|  |  | PR139: TTAGGATCCCCGTCTGTTCCTCCCTCTCG (Bam HI) |  |
| *KAT60c* | OL1566: GTCAGGATCCTCTCTTCAAAGCAAACGG (Bam HI) | PR11: GGAGACTAGTGACGACGACGTGGGGCTTC (Spe I) | OL1623: CGCGGGCCCATGGACGACGACGTGGGGCTTC (Apa I) |
|  | OL1567: GGGAAGCTTATGTTTTTGCCCTGTGGAAG (Hind III) | PR12: CTGCCTCGAGTGCCCTGTGGAAGAGGCGC (Xho I) | OL1624: CGCGGATCCCTACAGATCTTCTTCAGAAATAAGTTTTTGTTCCACTGACCCCAGCTCTTCCGC (Bam HI) |
|  |  | PR13: GGAACTCGAGGGAGGAAGAGATGTATAG (Xho I) |  |
|  |  | PR14: GTGTGGATCCTGGTAAAAACCCTTTGCAGCC (Bam HI) |  |
| *SPA* | PR78: ATAGGATCCGCCCGTGAGTTGATTGCCTC (Bam HI) | PR122: TTATCTAGAGGTCGCCACCATGATGTGTC (Xba I) |  |
|  | PR79: TATCTCGAGCGTCAGGACTCTCGTCATCA (Xho I) | PR123: TAATCTCGAGGCCGCATTTAAGGAGCACAG (Xho I) |  |
|  |  | PR120: TCTCGAGGTGTAGGTAGCTTTACCTTCAC (Xho I) |  |
|  |  | PR121: AGGATCCGTAGAAACGAAATTCCCGTCTC (Bam HI) |  |
| *FID* | PR73: ATAGGATCCGAAGACCGAGTTTCTCGTGC (Bam HI) |  |  |
|  | PR74: TATCTCGAGTCCATTCCACATAACGACGA (Xho I) |  |  |
| *c-myc:*  *mCherry* |  | PR7: GAGAAAGCTTATGGAACAAAAACTTATTTCTGAAGAAGATCTGACTAGTGCAACTAGCGGCATGGTTAG (Hind III, Spe I) |  |
|  |  | PR29: GGAGTCTAGATGCGGTACCAGAACCTTTG (XbaI) |  |
| *ha* |  | PR9: AGCTTTACCCTTACGATGTGCCTGATTACGCGTACCCATACGACGTGCCAGACTACGCATACCCGTACGATGTGCCCGATTACGCAA |  |
|  |  | PR10: CTAGTTGCGTAATCGGGCACATCGTACGGGTATGCGTAGTCTGGCACGTCGTATGGGTACGCGTAATCAGGCACATCGTAAGGGTAA |  |
